# Hepatitis B virus particles activate B cells through the TLR2–MyD88–mTOR axis

**DOI:** 10.1038/s41419-020-03284-1

**Published:** 2021-01-04

**Authors:** Qian Li, Jun Wang, Heba Islam, Carsten Kirschning, Hongzhou Lu, Daniel Hoffmann, Ulf Dittmer, Mengji Lu

**Affiliations:** 1 Institute of Virology, University Hospital of Essen, University of Duisburg-Essen, Essen, Germany; 2 Department of Infectious Diseases, Shanghai Public Health Clinical Center, Shanghai, China; 3 Center of Clinical Laboratory, The Fifth People’s Hospital of Wuxi, Jiangnan University, Wuxi, Jiangsu China; 4 Bioinformatics and Computational Biophysics, University of Duisburg-Essen, Essen, Germany; 5 Institute of Medical Microbiology, University Hospital of Essen, University of Duisburg-Essen, Essen, Germany

**Keywords:** Viral infection, Pattern recognition receptors

## Abstract

Host immune control plays a pivotal role in resolving primary hepatitis-B-virus (HBV) infections. The complex interaction between HBV and host immune cells, however, remains unclear. In this study, the transcriptional profiling of specimens from animals infected with woodchuck hepatitis virus (WHV) indicated TLR2 mRNA accumulation as most strongly impacted during WHV infection resolution as compared to other mRNAs. Analysis of blood transcriptional modules demonstrated that monocytes and B-cells were the predominantly activated cell types in animals that showed resolution of infection, which was similar to the response of TLR2-stimulated PBMCs. Further investigation of TLR2-stimulated B-cells pointed at interactions between activated TLR signaling, Akt-mTOR, and glucose metabolic pathways. Moreover, analysis of B-cells from Tlr2^−/−^, Trif^−/−^, Myd88^−/−^, and Trif/Myd88^−/−^ mice challenged with HBV particles indicated B-cell function and glucose metabolism alterations is TLR2-MyD88-mTOR axis dependent. Overall, our study implicates B-cell TLR2 activation in HBV infection resolution.

## Introduction

More than two billion people worldwide have been exposed to hepatitis B virus (HBV). Approximately, 240 million people are chronically infected with HBV, leading to more than 700,000 deaths per year because of hepatocellular carcinoma and cirrhosis^1^. In immunocompetent adults, however, HBV typically causes transient self-limited hepatitis B infection^2^. Primary HBV infection is controlled by a complex and concerted response of host innate, cellular, and humoral immunity. The interactions involving these immunological processes remain unclear. Recently, the activation of host immune cells by Toll-like receptors (TLRs) has been studied using different experimental systems.

The HBV nucleocapsid was once considered to be a TLR2 ligand that stimulates macrophages to produce pro-inflammatory cytokines in vitro^3^. However, lipoprotein contamination of the TLR2/4 pathway originating from the bacterial purification system could not be excluded^4,5^. Our recent studies indicated TLR2 as a contributor to T cell-dependent hydrodynamic HBV infection clearance in mice and primary human hepatocyte recognition of nascent HBV particles by TLR2 towards the activation of anti-HBV immune responses in vitro which challenges the former classification of HBV as “stealth” virus^6,7^. Moreover, impacting of TLR activation on the glycolytic metabolism drives macrophage and T-cell activation, suggesting TLR regulation of mTOR-integrated immune and metabolic processes^8–13^.

Recently, defective circulating and intrahepatic antiviral B-cell responses in patients with hepatitis B have gained attention^14,15^. The diverse antiviral roles of B cells, including humoral immunity, antigen presentation, and cytokine production may be disabled in persistent viral infections^16–18^. A recent study also showed the supporting role of metabolic reprogramming in naïve B-cell activation for effective immune response^19^. However, how TLR2 contributes to B-cell activation and metabolism against HBV infection remains unclear. In this study, we examined whether and how B cells respond to cell culture-derived HBV particles in vitro.

## Results

### TLR2 contributes mainly to resolving woodchuck hepatitis virus infection

**Fig. 1:**
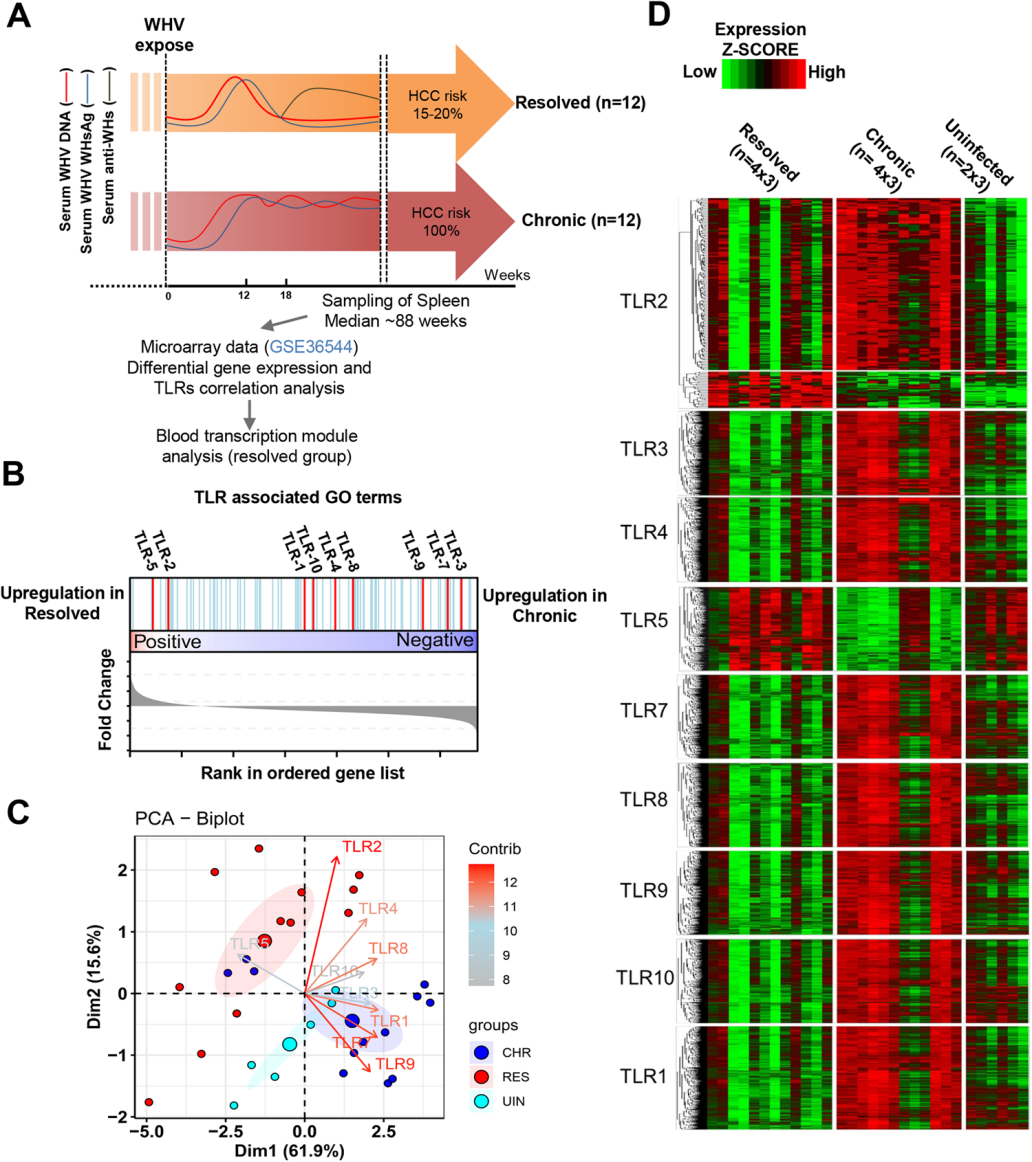
TLR2 contributes mainly to the resolution of WHV infection. **A** All resolved WHV animals were WHV DNA-undetectable. Chronically infected animals were all anti-WH negative, with serum WHV DNA levels ≥10^10^ ge/mL. **B** All genes in TLR-associated GO terms were compared between resolved WHV and chronic WHV samples. **C** PCA was performed with R package “factoextra” to visualize the contribution of all TLRs to the outcome of WHV infection. **D** TLR-related DEGs are shown in a scaled heatmap with a comparison of resolved WHV (*n* = 4), chronic WHV (*n* = 4), and uninfected samples (*n* = 2). Each group includes three technical replicates.

Our previous studies suggested the involvement of TLRs in immune control of HBV infection and clearance. However, the role of distinct TLRs in natural HBV infection is yet unclear. We explored the published transcriptome data (GSE36544) from the woodchuck model to assess the relative contribution of TLRs in self-limiting and chronic woodchuck hepatitis virus (WHV) infection^20^. We monitored all animals for one year after infection and assigned them to infection status groups using specific serological markers (WHV DNA, WHsAg, and anti-WH surface antibody) as shown in Fig. 1A.

We analyzed differential gene expression with Limma to compare the spleen signature between resolved WHV and chronic WHV samples (*p* value < 0.05). TLR-associated GO terms are shown in Fig. 1B. The results indicated that the expression of TLR2 and TLR5 was upregulated in animals with resolved WHV infection compared with chronically WHV-infected woodchucks (Fig. 1B).

We performed principal component analysis (PCA) with R-package “factoextra” to visualize the contribution of all TLRs to the outcome of WHV infection. The data showed that TLR2/5 contributed to resolving WHV infection, whereas TLR1/7/9 contributed mostly to chronic WHV infection (Fig. 1C).

TLR1-10-related (Pearson correlation, *R* value > 0.5 and *p* value < 0.05) DEGs were displayed in a scaled heatmap toward a comparison of resolved WHV infection with chronic WHV infection and uninfected controls. As shown in Fig. 1D, a set of TLR2-related genes was upregulated in resolved WHV infection. In contrast, TLR5-related genes were downregulated in chronic WHV infection.

### B cells and macrophages but not T cells are immune cell types related to TLR2 response in resolved WHV infections

**Fig. 2:**
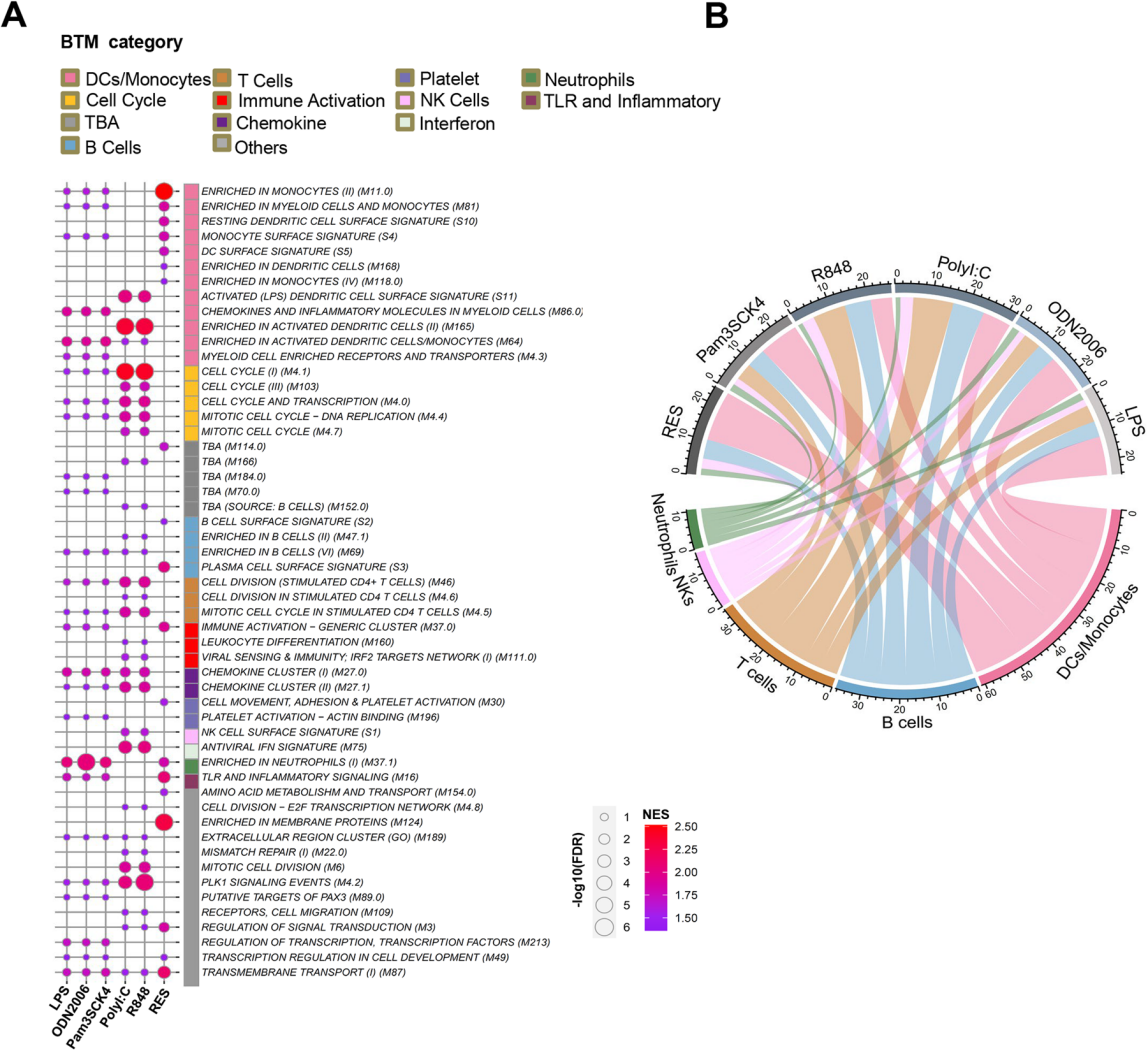
Translation of gene expression patterns to specific immune functions in resolved WHV. **A** Gene enrichment analysis was used in BTMs. GSEA of a pre-ranked gene list according to correlated *R* value with TLR2 and fold-change of TLR2-stimulated PBMCs. All enriched modules (>10 genes, FDR < 0.25) are listed. **B** The sum of normalized enrichment scores of modules in specific cell-types was calculated and presented using a chord diagram.

To translate gene expression patterns to specific immune functions in resolved WHV animals, we used BTMs as gene sets to perform GSEA. BTMs were previously established from more than 30,000 human blood transcriptomes obtained from more than 500 studies in public databases^21^. Each BTM contained a set of genes with correlated expression patterns and annotated with similar biological functions. We performed GSEA on a pre-ranked gene list according to the fold-change of mRNA expressions in TLR2/3/4/7/8/9-activated peripheral blood mononuclear cells (PBMCs) challenged with bacterial protein analog P3C, ssRNA analog PolyI:C, bacterial LPS, dsRNA analog R848, an oligodeoxynucleotide (ODN) 2006, respectively. We also performed GSEA based on a pre-ranked gene list according to the fold change in woodchucks having resolved WHV infection compared to uninfected controls. The normalized enrichment scores of modules for specific cell-types were displayed in detailed BTMs (Fig. 2A). In addition, the modules including TLR pathways and inflammatory, immune activation, and cell cycle-related modules were also enriched (Fig. 2A).

Then, we calculated the sum of normalized enrichment scores of the modules for specific cell-types to define which TLR stimulation was similar to RES module enrichments (Fig. 2B). According to the enriched modules for the immune cell types, DC/Monocytes and B cells were found to be the cell types most remarkable in resolved WHV infection (RES), a result which was similar to the response of P3C-stimulated PBMCs with obviously higher proportions of DCs/Monocytes and B cells (Fig. 2B).

We further compared the gene signatures by using fold change data (Log_2_FC) of woodchucks (fold change of mRNA expression in woodchucks with resolving WHV infection compared to uninfected controls) to TLR-stimulated B cells, T cells, and macrophages, respectively (fold change of mRNA expression in TLRs-stimulated cells compared to unstimulated controls) (Fig. S1). The fold changes of selected genes in resolved WHV infection was similar to macrophages and B cells, but not T cell (Fig. S1). Previously, it has been reported that TLR2 induces metabolic reprogramming in macrophages^12,13^, thus, we focused on metabolism related TLR2 activity of B cells in this study.

### Activation of B cells TLR2 impacts the Akt-mTOR pathway and increases glucose metabolism

**Fig. 3:**
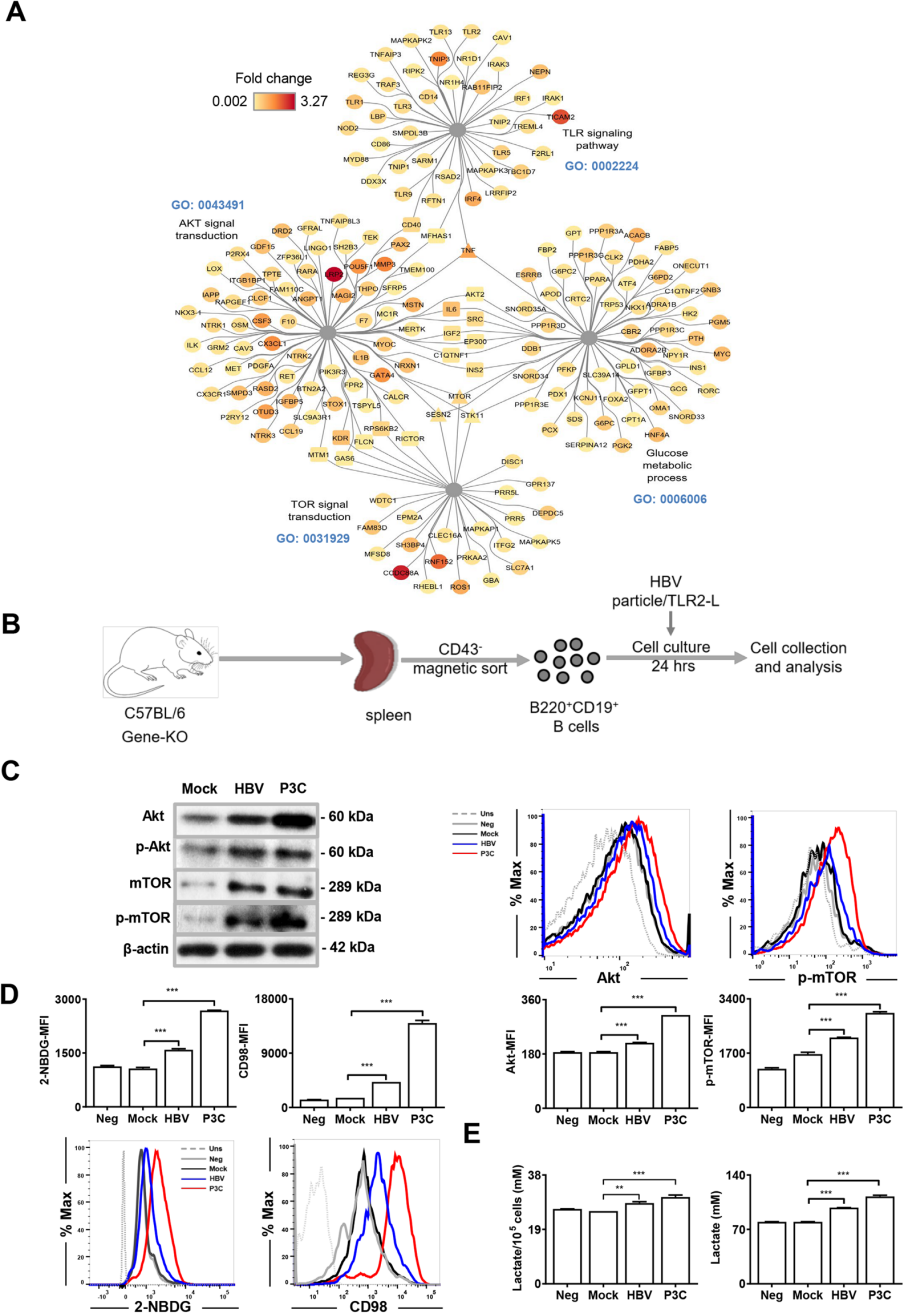
TLR2 interacts with Akt-mTOR pathway and glucose metabolism. **A** Interaction network between TLR, Akt, mTOR, and glucose metabolism. **B** Sorting scheme for isolating naïve mouse B cells and in vitro stimulation. **C** Expression of Akt and p-mTOR in HBV particles/TLR2-stimulated B cells were detected by Flow cytometry and Western blotting. **D** Glucose and glutamine uptake in HBV particles/TLR2-L stimulated B cells was measured by detecting the MFI of the glucose analog 2-NBDGs and CD98. **E** Lactate production in the cell culture medium in HBV particle/TLR2-L-stimulated B cells was detected with specific kits. Data are representative of two independent experiments. All data are presented as mean ± SD (*p < 0.05; **p < 0.01; ***p < 0.001) statistical relevance was determined by one-way ANOVA.

Our previous study demonstrated that TLR stimulation enhanced T-cell function by increasing cellular glycolysis. The mTOR signaling pathway interacts with innate immunity and plays a vital role in regulating cellular glycolysis in TLR2 and -7-activated CD8+ T cells^9,10^. Thus, we hypothesized that TLR2 activation of B cells would be associated with glucose metabolism, as energy supply is essential to upregulated cellular processes. We explored the microarray data of GSE28517 to identify upregulated genes in TLR2-stimulated mouse B cells^22^. We ranked the selected 196 genes in glucose metabolic process (GO term: 0006006), Akt signal transduction (GO term: 0043491), TLR signaling pathway (GO term: 0002224), and TOR signal transduction (GO term: 0031929) by log (fold-change) (Fig. 3A) and integrated them using Cytoscape software (version: 3.8.0). Within the TLR signaling pathway, *TLR2*, *MyD88*, *IRAK*, and *CD86* mRNAs were upregulated. According to the virtual interaction network we applied, the TLR pathway was associated to the Akt pathway by *MFHAS1*, *TNF*, and *CD40*. The Akt pathway was involved in glucose metabolism through the *INS2*, *EP300*, *SRC*, *AKT2*, *IL6*, *TNF*, *IGF2*, *C1QTNF3*, *MTOR*, *SESN2*, and *STK11* genes. The mTOR pathway was linked to glucose metabolism by the *MTOR*, *SESN2*, and *STK11* genes. Genes related to glucose metabolism, such as *HK2*, *G6PC*, *G6PD2*, and *FBP2*, participated in glycolysis (Fig. 3A).

In total 3958 TLR2-related mRNA expression changes were validated as being involved in the resolution of WHV infection and the products of 196 genes mRNAs were upregulated under this condition were involved in four GO terms, namely glucose metabolic process: 0006006, Akt signal transduction: 0043491, TLR signaling: 0002224, and TOR signal transduction: 0031929 were also upregulated after TLR2 activation (Fig. S2). We constructed a Venn diagram (Fig. S2) to show the number of equally or distinctly induced genes, with 29 common genes. Interestingly, glucose metabolism-related genes, such as *HK2*, *PFKP*, *MYC*, and *IRF4*, were involved in the 29 shared genes. Moreover, *mTOR* and *MyD88* also were included in the 29 shared genes.

Our previous studies showed that exposure of hepatic cells including hepatocytes, liver sinusoidal cells, and Kupffer cells to HBVs or the canonical TLR2 ligand P3C resulted in diverse cellular responses^6,23–26^. Thus, now we asked whether and how exposure of B cells to HBVs changes their activation status. We next performed in vitro experiments to investigate whether TLR2 activation acted through Akt-mTOR signaling in B cells (Fig. 3B). Akt and mTOR expression and phosphorylation were detected by immunoblotting and flow cytometry. The results showed that TLR2 agonist (P3C) and HBV particles (HBVs) stimulation increased the levels of Akt, m-TOR, phosphorylated Akt, and phosphorylated mTOR in B cells (Fig. 3C). We further examined whether TLR2 activation induced metabolic changes. We measured glucose uptake and glutamine transport by detecting the mean fluorescence intensity (MFI) of the glucose analogs 2-NBDG and CD98, respectively. We observed significantly increased MFI of 2-NBDG and CD98 in B cells at 24 h upon P3C and HBVs stimulation (Fig. 3D). Furthermore, higher production of lactate in the culture supernatants by aerobic glycolysis was observed after 24 h stimulation with P3C and HBVs (Fig. 3E). Thus, TLR2 activation induced Akt-mTOR activation and elevated glucose metabolism, particularly glycolysis in B cells.

### HBV particles and P3C driven B-cell function

**Fig. 4:**
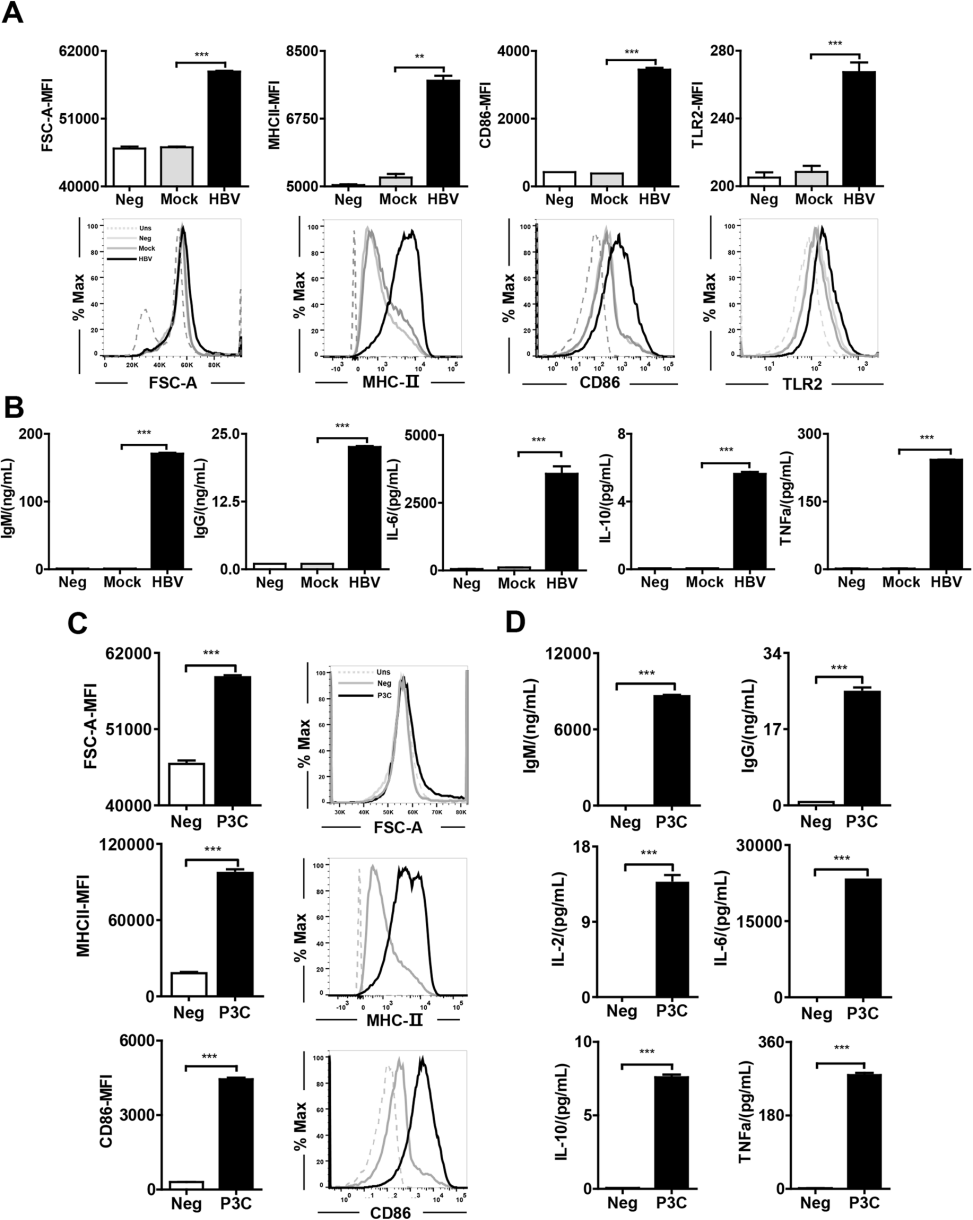
HBVs and TLR2-L promote B-cell function. **A** FSC-A, MHC II, CD86, and TLR2 expression in HBV-stimulated B cells was measured by flow cytometry. **B** Antibodies, including IgG, IgM, and cytokine production, including IL-6, IL-10, and TNF-α, in HBV-stimulation, were measured by ELISA. (**C**) FSC-A, MHC II, and CD86 expression in TLR2-stimulated B cells were measured by flow cytometry. **D** Antibodies, including IgG and IgM, and cytokine production, including IL-6, IL-10, and TNF-α, in TLR2-L-stimulated B cells was measured by ELISA. Data are representative of three independent experiments. All data are presented as the mean ± SD (*p < 0.05; **p < 0.01; ***p < 0.001) according to *t* test (**A**, **B**) and one-way ANOVA (**C**, **D**).

To test the direct effect of HBV on B cells, we used the in vitro assay as described (Fig. 3C). We purified B cells from naïve mice to >96% (Fig. S3A) and stimulated the cells with HBVs at different multiplicities of infection (MOIs = 200–1000) in vitro. Compared with the mock control, HBVs at MOI: 1000 increased cell viability (Fig. S3B) and cell activation with a maximum effect (Fig. S3C). After 24 h of exposure with HBVs (MOI = 1000), flow cytometry analysis showed that the cell size of B cells increased as measured by forward scatter (FSC-A). In addition, HBVs potently upregulated the expression of TLR2 and activation markers, such as MHC II and CD86, in B cells (Fig. 4A). The secretion of IgM and IgG antibodies and cytokines, including IL-6, IL-10, and TNF-α, was also enhanced (Fig. 4B). Interestingly, TLR2 expression on B cells was upregulated after HBVs exposure (Fig. 4A).

In parallel, we used P3C (0.01–10 μg/mL) to stimulate B cells in vitro. A dose of 2 μg/mL of P3C (Fig. S3D) was selected for further experiments because of the observed maximal stimulation of MHC II and CD86 expression in B cells. Except for the elevated MHC II and CD86 expression, B-cell size as measured by FSC-A, the secretion of IgM and IgG antibodies, and cytokines (such as IL-2, IL-6, IL-10, and TNF-α) were increased also upon P3C challenge of B cells (Fig. 4C, D).

### AKT-mTOR signaling and glucose metabolism mediate TLR driven enhancement of B cell activity

The AKT/mTOR pathway plays a major role in mediating TLR activation to the downstream processes, including cellular metabolism^9,13^. Thus, we investigated the involvement of the AKT/mTOR pathway in TLR2-mediated B-cell activation.

**Fig. 5:**
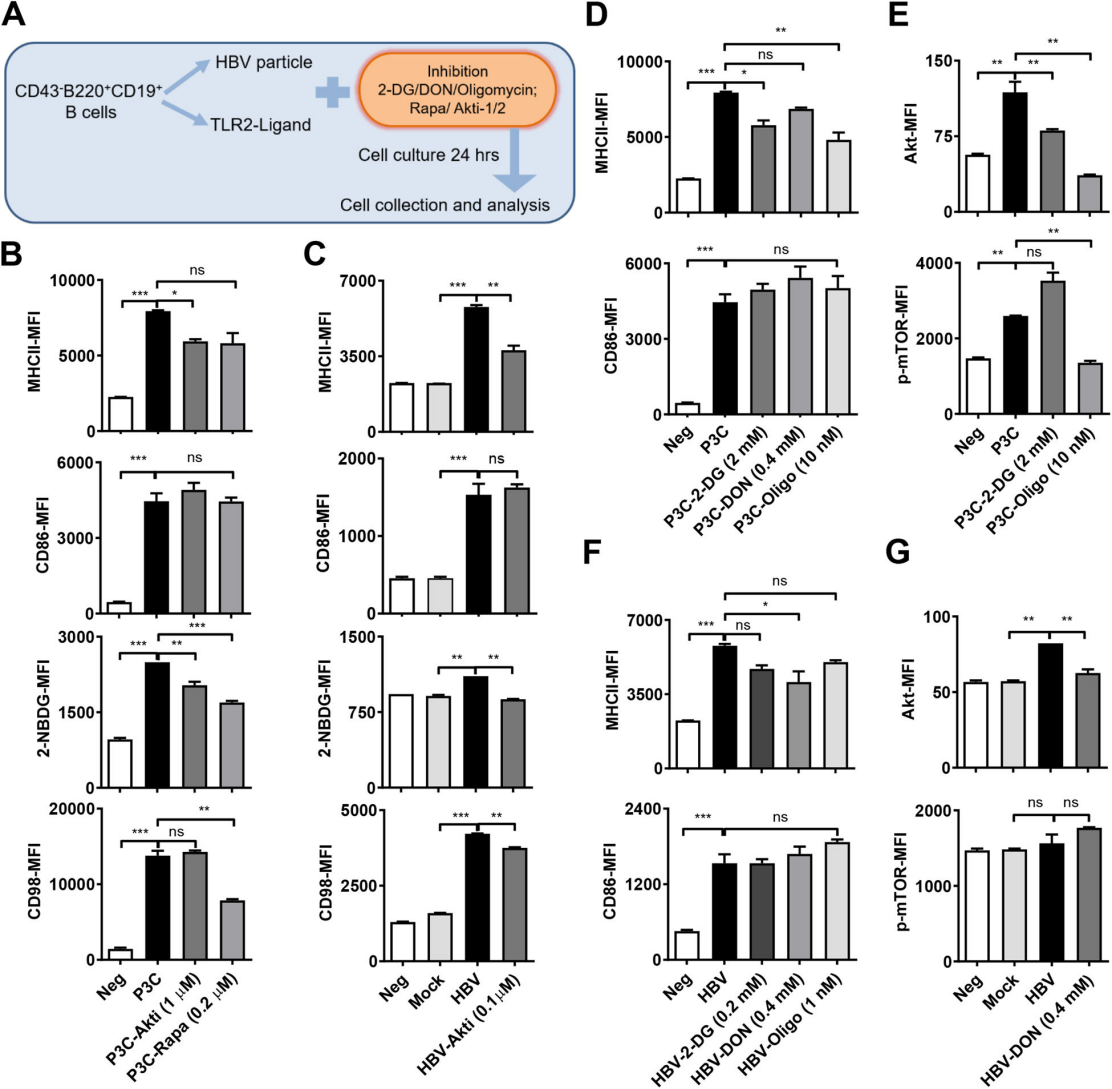
Blocking the Akt-mTOR and metabolic pathways in HBV/TLR2-L stimulates B cells. **A** Purified B cells were stimulated with TLR2-L/HBV particles for 24 h with or without metabolic pathway inhibitors. **B**, **C** MHC II and CD86 expression in B cells was detected by flow cytometry following treatment with Rapamycin/Akti-1/2. Glucose and glutamine uptake was measured by detecting the MFI of the glucose analog 2-NBDG and glutamine transporter CD98 following treatment with Rapamycin/Akti-1/2. **D**, **F** MHC II and CD86 expression in B cells was detected by flow cytometry following metabolic blocking. **E**, **G** Akt, and p-mTOR expression were detected by flow cytometry following treatment with 2-DG/DON/oligomycin. Data are representative of three independent experiments. All data are presented as the mean ± SD (*p < 0.05; **p < 0.01; ***p < 0.001) according to one-way ANOVA.

We treated TLR-activated B cells with two different AKT/mTOR pathway inhibitors: Rapamycin and Akti-1/2 (Fig. 5A). The viability of P3C- (Fig. S4A) and HBV- (Fig. S4B) stimulated B cells did not decrease at the indicated doses of Rapamycin and Akti-1/2. Therefore, we employed Rapamycin (0.2–5 μM) and Akti-1/2 (0.1–1 μM) in further inhibition experiments of TLR2-stimulated B cells; we selected Akti-1/2 (0.1 μM) for inhibition experiments of HBV-stimulated B cells. Inhibition of mTOR slightly reduced P3C- (Fig. 5B) and HBV-induced (Fig. 5C) B-cell activation, as indicated by the lower number of B cells expressing the activation marker MHC II. We also asked whether TLR2-induced mTOR activation depends upon metabolic changes. The optimum dose of Rapamycin (0.2 μM) decreased P3C-improved glucose uptake and glutamine transport, indicated by a drop of 2-NBDG and CD98 MFIs, respectively (Fig. 5B). A 0.1 μM Akti-1/2 counteracted HBV and P3C induced 2-NBDG uptake and CD98 expression (Fig. 5C). Thus, the AKT/mTOR signaling pathway mediates HBV/P3C-elevated B cell function and metabolism.

We treated B cells with three different metabolic pathway inhibitors, namely 2-DG, oligomycin, and DON. The results indicated that despite stimulation with HBVs or P3C (Fig. S4A, B), inhibition of the metabolic pathway reduced the number of vital B cells. However, we found that at a specific dose of metabolic inhibitors, the number of vital B cells was not increased. This dose of metabolic inhibitors slightly decreased the expression of MHC II. Among these, 2-DG decreased the expression of MHC II and Akt in P3C-stimulated B cells. Oligomycin decreased the expression of MHC II, Akt, and phosphorylated mTOR in P3C-stimulated B cells (Fig. 5D, E). DON inhibition significantly decreased the expression of MHC II and Akt in HBV-stimulated B cells (Fig. 5F, G).

Taken together, these data show that the Akt–mTOR pathway is TLR-driven and enhances immune functions of B cells, which also affects/involves metabolism.

### HBV particles enhance the function and metabolism in B cells via MyD88

TLR activation requires downstream adaptors for signal transduction. MyD88 and TRIF are two major components of the cascade^27^. We investigated whether these two proteins participated in TLR2-mediated B-cell activation.

**Fig. 6:**
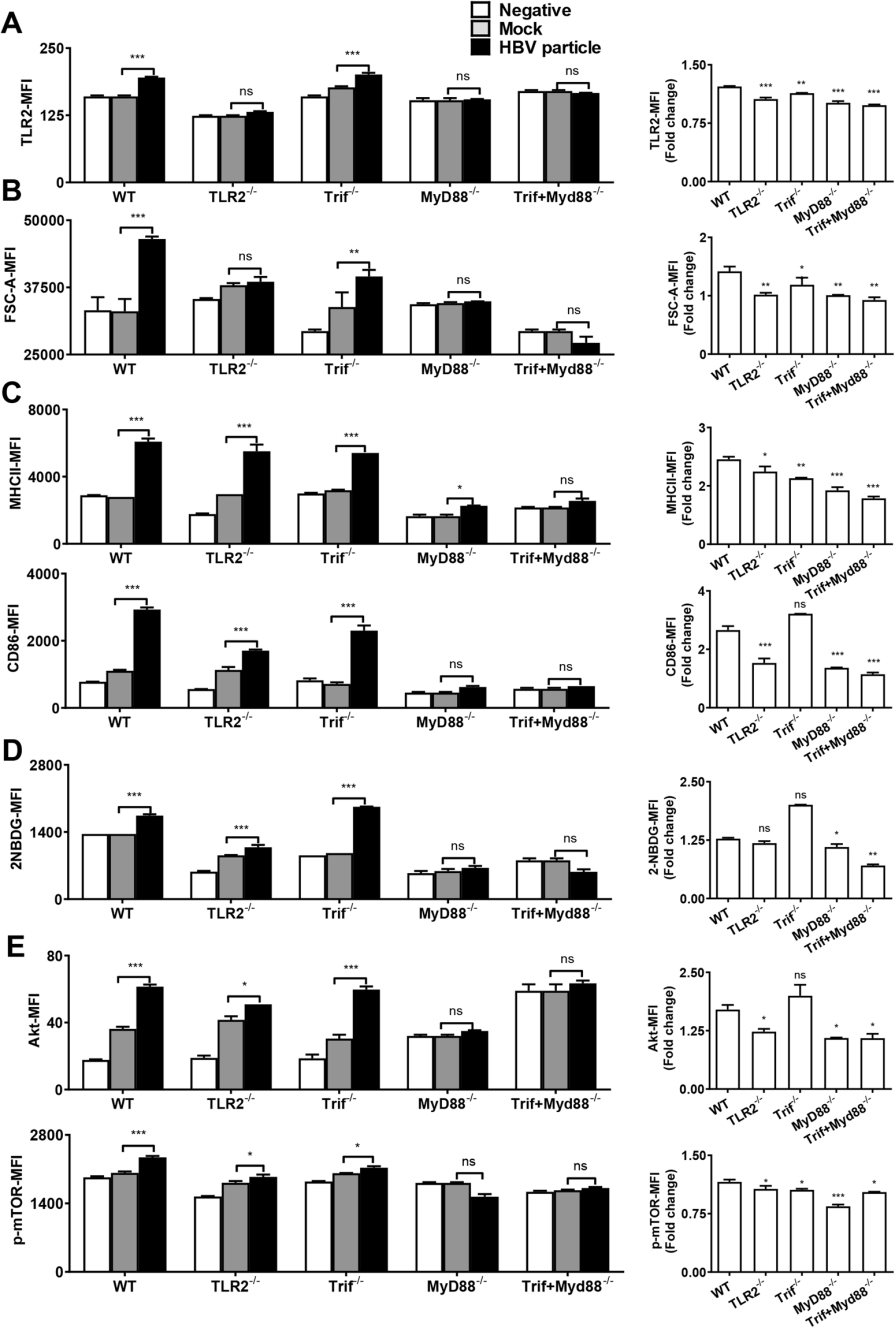
HBV-enhanced B-cell function and metabolism are MyD88-dependent. Purified B cells were derived from WT, TLR2^−/−^, TRIF^−/−^, MyD88^−/−^, and TRIF/MyD88^−/−^ mice and stimulated with HBV particles for 24 h. **A** TLR2 expression in B cells was detected by flow cytometry in WT, TLR2^−/−^, TRIF^−/−^, MyD88^−/−^, and TRIF/MyD88^−/−^ B cells. **B**, **C** B-cell size and activation were assessed by FSC-A, MHC II, and CD86. **D** Glucose uptake was measured by detecting the MFI of the glucose analog 2-NBDG in WT, TLR2^−/^^−^, TRIF^−/−^, MyD88^−/−^, and TRIF/MyD88^−/−^ B cells after 24 h of stimulation. **E** Akt and p-mTOR expression were detected by FACS in WT, TLR2^−/−^, TRIF^−/−^, MyD88^−/−^, and TRIF/MyD88^−/−^ B cells after 24 h of stimulation (*p < 0.05; **p < 0.01; ***p < 0.001). Data are representative of three independent experiments. All data are presented as the mean ± SD (*p < 0.05; **p < 0.01; ***p < 0.001) according to two-way ANOVA.

To confirm that TLR2-MyD88 signaling is required for the regulation of B-cell activation following HBV stimulation, we stimulated B cells derived from WT, TLR2^−/−^, TRIF^−/−^, MyD88^−/−^, or TRIF/MyD88^−/−^ mice with HBVs. The results indicated that upon stimulation of HBVs, TLR2 expression increased only in B cells derived from WT and TRIF^−/−^ mice but not in B cells derived from TLR2^−/−^, MyD88^−/−^, or TRIF/MyD88^−/−^ mice (Fig. 6A, left panel). The size of the B cells assessed by FSC-A increased only in WT and TRIF^−/−^ mice, but not in TLR2^−/−^, MyD88^−/−^, and TRIF/MyD88^−/−^ mice (Fig. 6B, left panel). Fold change in the MFI was calculated with respect to unstimulated controls. The fold change of TLR2 expression and FSC-A was obviously decreased in TLR2^−/−^, TRIF^−/^^−^, MyD88^−/−^ and TRIF/MyD88^−/−^ mice compared to WT mice (Fig. 6A, B, right panel). The expression of MHC II was obviously increased in WT, TLR2^−/−^, and TRIF^−/−^ mice but not, or only slightly increased in MyD88^−/−^ and TRIF/MyD88^−/−^ mice. The expression of CD86 in B cells was increased in WT, TLR2^−/−^, and TRIF^−/−^ mice but not in MyD88^−/−^ and TRIF/MyD88^−/−^ mice (Fig. 6C, left panel). The fold changes of MHC II and CD86 expression were significantly decreased in MyD88^−/−^ and TRIF/MyD88^−/−^ mice as compared to other groups (Fig. 6B, C, right panel). In turn, HBV stimulation increased the uptake of 2-NBDG and expression of Akt and p-mTOR in B cells from WT, TLR2^−/−^, and TRIF^−/−^ mice, but not from MyD88^−/−^ and TRIF/MyD88^−/−^ mice (Fig. 6D, E, left panel). The fold changes of metabolism parameters such as 2-NBDG, Akt, and p-mTOR were significantly lower in MyD88^−/−^ and TRIF/MyD88^−/−^ mice as compared to other groups (Fig. 6D, E, right panel). Thus, the mTOR signaling pathway plays an important role in mediating the TLR2-induced elevation in glycolytic metabolism in CD8+ T cells. These results showed that TLR2-MyD88 was required for HBV-mediated enhancement of immune functions and metabolism in B cells.

These findings indicated that after stimulation with HBV particles, MyD88-deficient B cells abolished HBV-mediated TLR expression and effector functions, confirming that the process depended on the MyD88 pathway.

## Discussion

One of the main issues in the field of HBV research is the role of TLRs in HBV infection. The present study analyzed whether TLR2 mostly contributed to resolving WHV infection. Only TLR2-related genes were upregulated in resolved WHV samples. To investigate which cell type was mostly regulated by TLR2 activation in the antiviral response, we used BTMs to analyze the spleen signature of resolved WHV and TLR2-stimulated PBMCs. The results revealed that TLR2 activation was related to the enrichment of monocytes and B cells. Furthermore, by comparing TLR2-stimulated T/Mac/B cells to resolved spleen samples, we found TLR2-activated B cells to be more similar to the signature of resolved WHV spleen samples.

Zhang et al. found that HBVs induce an immune response to TLR2 signaling in the primary human hepatocytes, suggesting that HBV is a cunning but not stealthy virus^6^. Our in vitro findings demonstrated that HBV also activated the innate immune signaling pathway (TLR2) in B cells. Both HBVs and TLR2 stimulation promoted B-cell activation, suggesting that HBV-induced B-cell activation by TLR2 is a factor contributing to the resolution of HBV infection.

Early studies on TLR activation in monocytes and T cells indicated that TLR-enhanced cellular function was regulated by metabolic processes. The relationship between HBV particle-stimulated TLRs and metabolic function in B cells, however, remains unclear. Our transcriptome analysis of TLR2-stimulated B cells revealed a potential interaction among TLRs, Akt, mTOR, and glucose metabolism pathways. To further investigate the interaction of TLR2 with the mTOR pathway, we detected Akt/mTOR expression in B cells and found that HBV and P3C upregulated both the expression and phosphorylation of Akt/mTOR. Studies on metabolic pathways are needed to confirm B-cell activation^28,29^. Stimulation of B cells with lipopolysaccharide, a TLR4 receptor agonist, or an anti-IgM antibody to cross-link the B-cell antigen receptor increased glucose import into activated B cells and increased the oxygen consumption rate^30–32^. Along with the enhanced function in B cells, our results indicated that HBVs and P3C increased glucose uptake and lactate production.

Previous studies have shown that mTOR regulates important cellular processes, such as cell survival, metabolism, and autophagy^18,33,34^. mTOR signaling can induce complex networks of reprogramming, including enhanced aerobic glycolysis, to facilitate rapid clonal expansion. Interestingly, in our study, blocking the AKT-mTOR signaling pathway with Akti-1/2 or Rapamycin impaired the enhanced function and metabolism in HBV/P3C-activated B cells. To further elucidate the role of metabolic pathways in HBV/P3C-stimulated B cells, we applied different metabolic inhibitors and found them to block B-cell functions. The blocking of metabolism also may have decreased Akt and mTOR phosphorylation, indicating an interaction between the mTOR pathway and metabolism in B cells.

TLRs recognize distinct pathogen-associated molecular patterns and attract adaptor proteins, such as TRIF and MyD88, facilitating the further accumulation of kinase-like IL-1 receptor-associated kinase-4 for signal transduction^27^. In our study, we observed eliminated B-cell activation, glucose uptake, and Akt and p-mTOR expression in MyD88^−/−^ and TRIF/MyD88^−/−^ mice, whereas these processes were not blocked in TLR2^−/−^ or TRIF^−/−^ mice in the presence of HBVs. MyD88 is required in B cell activation after treated with HBV particle, but not TLR2 only. MyD88 is the downstream adaptor molecule of several TLRs such as TLR1/6, TLR2, TLR4, TLR7, TLR9, while TLR2 was only expressed on the cell surface.

**Fig. 7:**
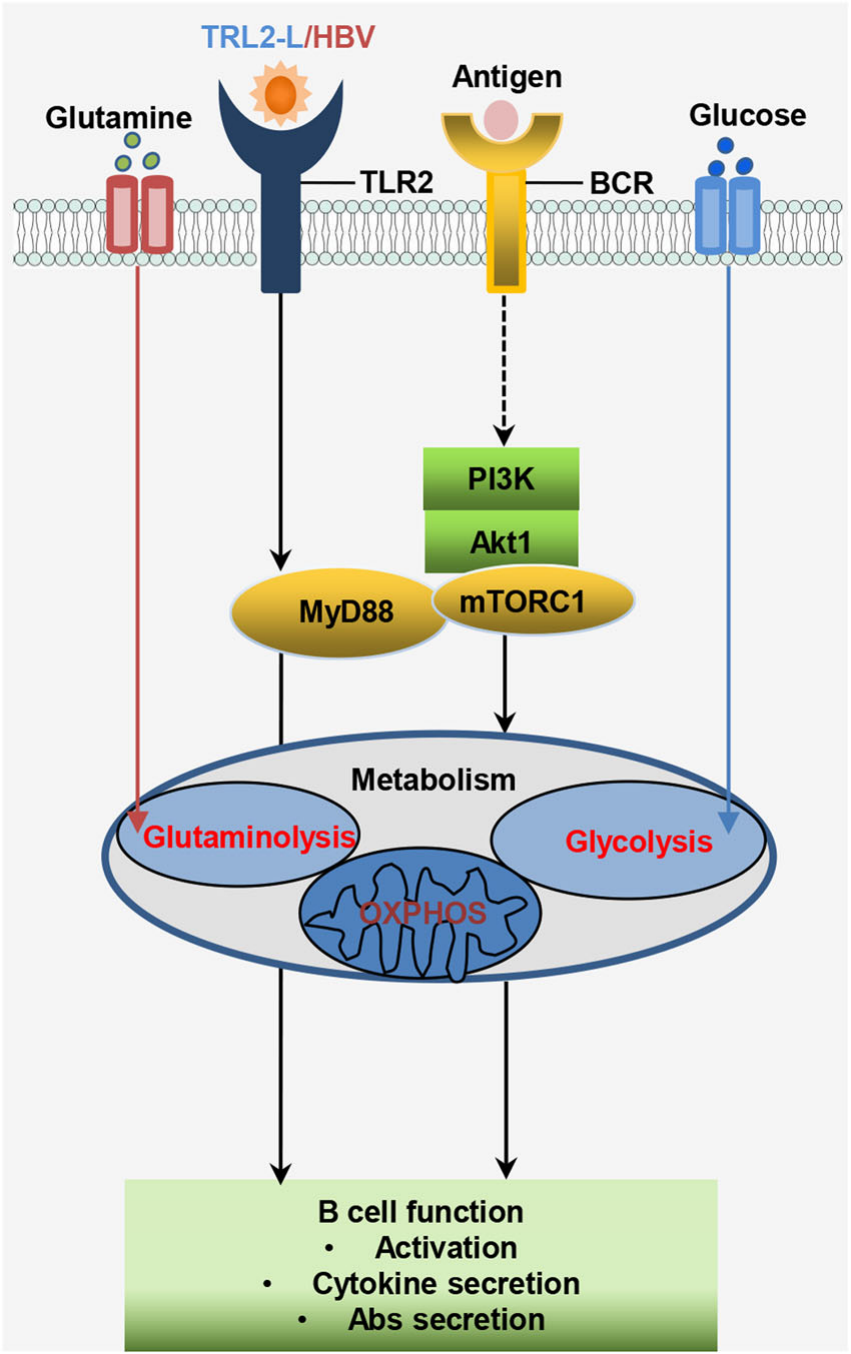
Schematic diagram of HBV/TLR2-mediated B-cell activation. HBV/TLR2 stimulation promotes the activation of B cells. TLR2-mediated B-cell activation relies on MyD88-AKT-mTOR signaling, leading to metabolic reprogramming of B cells, including the upregulation of glucose uptake and glutamine transport, which in turn activate B cells. Signaling pathways identified in this study are indicated by solid arrows. Potential signaling pathways are represented by broken arrows.

In conclusion, the results of this study suggested that TLR2 was the only TLR involved in WHV resolution and that HBV particles were recognized by TLR2 to initiate B-cell activation in vitro. Moreover, a virus-host interplay occurred in B cells through a coordinated balance between cell activation and metabolism that involved immune induction during HBV infection. These observations highlighted that HBVs induced B-cell activation and metabolism through the TLR2–MyD88–mTOR pathway (Fig. 7). Further studies are needed to determine the underlying mechanisms at the single-cell level, taking into account spatial and temporal events, which may lead to the development of novel treatments for chronic HBV.

## Materials and methods

### Data and samples

We used gene expression in the spleens of uninfected, resolved, and chronically infected woodchucks in microarray analysis. The results were deposited in NCBI’s Gene Expression Omnibus, which is accessible through GEO series accession number GSE36544^20^. Gene expression in TLR2- stimulated PBMCs is available through GEO series accession number GSE131818^35^. Gene expression in TLR2-stimulated purified B cells, T cells, and macrophages is available through GEO series accession numbers GSE28517, GSE89513, and GSE81291, respectively^22,36,37^.

### Mice

We obtained C57BL/6 mice (wild-type, WT mice) from Harlan Winkelmann Laboratories (Borchen, Germany). Deficient mouse strains, including TRIF^−/−^, TLR2^−/−^, MyD88^−/−^, and TRIF/MyD88^−/−^ mice, were bred under specific pathogen-free conditions at the Laboratory Animal Facility of the University Hospital Essen, Essen, Germany. All mice used for the experiment were 6–8 weeks of age. All mice were handled according to the Guide for the Care and Use of Laboratory Animals with approval from the district government of Düsseldorf, Germany.

### Microarray analysis

We acquired the normalized expression microarray data of each sample from GEO as earlier described. Probe sets without gene symbol annotation were omitted. For multiple probes targeting the same genes, we retained only the probe with the maximum mean. We used the linear model for microarray data (Limma) package, a modified *t*-test that integrates the Benjamini–Hochberg multiple hypotheses correction method, to assess differential expression genes^38^. Genes with a *p* value < 0.05 were defined as significantly differentially expressed.

### Principal component analysis

First, we applied hierarchical clustering (using Pearson’s correlation distance/average linkage method) in R version 3.6.1^39^. The top two principal components were displayed using the factoextra package for the mean point and confidence^40^.

### Ranked gene set enrichment analysis

We analyzed the differential regulation of blood transcriptome modules (BTMs)^21^ rather than those of individual genes. Each BTM includes a set of genes with correlated expression patterns and similar biological functions. The ranked gene set enrichment analysis (GSEA; threshold gene size at 10) was conducted according to the average fold-change between two groups^41^. The normalized enrichment score, false discovery rate, and gene size are shown in a bubble chart (Fig. 2A) prepared using the ggplot2 package.

### Gene ontology analysis

We focused our analysis on the terms Akt-signaling pathway (gene ontology [GO] term: 0043491), glucose metabolism pathway (GO term: 0006006), TLR signaling pathway (GO term: 0002224), and TOR signaling pathway (GO term: 0031929). We analyzed the interaction among different terms using Cytoscape software (version:3.8.0).

### B-cell isolation, culture, and activation in vitro

We prepared single-cell suspensions of mouse splenocytes as previously described^23^. We purified murine B cells from splenocytes using a B-cell isolation kit (Miltenyi Biotec, Bergisch Gladbach, Germany) according to the manufacturer’s guidelines.

We cultured B cells in RPMI complete medium (Thermo Fisher Scientific, Waltham, MA, USA). For murine B-cell activation, purified B cells were seeded into 96-well flat-bottom tissue culture plates at a density of 5 × 10^5^ cells/well with or without TLR2 ligand (P3C, 2 μg/mL; InvivoGen, San Diego, CA, USA)/HBV particles [Multiplicity of infection (MOI): 1,000] stimulation for 24 h. In some experiments, we treated the cells with the indicated inhibitors, such as 2-deoxy-d-glucose (2-DG), oligomycin, 6-diazo-5-oxo-l-norleucine (DON), Rapamycin, and Akti-1/2 (Sigma, St. Louis, MO, USA).

### HBV particle preparation

We cultured the cell line HepG2.117, stably transfected with a replication-competent HBV genome, as previously described^24^. We collected HBV particles (genotype D, serotype ayw, HBeAg-positive) from the cell culture supernatants, precipitated in 6% polyethylene glycol 8000 (PEG8000, Sigma) at 4 °C, and concentrated the supernatants by centrifugation (12,000*g*, 60 min, 4 °C). We produced a mock control by precipitating cell culture supernatants of HepG2 cells under the same conditions.

### Cell surface and intracellular staining

We performed cell surface and intracellular staining as previously described^25^. We conducted cell surface staining using antibodies, including αCD4 (clone GK1.5; Biolegend, San Diego, CA, USA), αCD19 (clone 6D5; Biolegend), αCD43 (clone S11; Biolegend), αB220 (clone RA3-6B2; Biolegend), αCD8 (clone 53-6.7; Biolegend), αTLR2 (clone QA16A01; Biolegend), and αCD98 (clone RL388; Biolegend).

For intracellular staining, the cells were permeabilized using a Cytofix/Cytoperm fixation and permeabilization solution kit (BD Biosciences, Franklin Lakes, NJ, USA) and stained with antibodies, including α-phospho-mTOR (clone MRRBY; eBioscience, San Diego, CA, USA) and αAkt (clone 55; BD Biosciences).

We detected stained cells using flow cytometry (LSR II, BD Biosciences) and conducted data analysis with FlowJo software (version 10.6.2; Ashland, OR, USA).

### Metabolism assays

We detected MFI of the glucose analog 2-NBDG (Thermo Fisher Scientific), which was represented in cells as glucose uptake. For 2-NBDG staining, we incubated cells at 37 °C in glucose-free media for 30 min. Then we added 200 μM 2-NBDG to glucose-free media for 20 min before cell surface staining. We measured lactate production using lactate colorimetric/fluorometric kit (Biovision, Milpitas, CA, USA). Glutamine transporter CD98 was stained using a specific antibody (clone MEM-108; Biolegend).

### Enzyme-linked immunosorbent assay

We measured cytokines (IL-2, IL-6, IL-10, and TNF-α) and antibodies (IgG and IgM) produced and secreted by B cells into the cell culture supernatants with enzyme-linked immunosorbent assay (ELISA) kits (Biolegend) according to the manufacturer’s instructions. We then measured the OD at a wavelength of 405 nm using an ELISA reader (Bio-Rad Model 550 l; Hercules, CA, USA).

### Statistical analyses

We performed data analyses using GraphPad Prism software (version 6, Inc.; San Diego, CA, USA). We compared data between two groups with a nonparametric t-test and those between more than two groups with a one-way ANOVA. For the experiments with immune-deficient mice, data were analyzed by a two-way ANOVA test. Significant differences between the two groups are indicated as follows: *p < 0.05, **p < 0.01, and ***p < 0.001. All results are representatives of two to three independent experiments.

## Supplementary information

Figure S1. Comparison of TLR2-related signature in specimens of animals

Figure S2. Overlapping of TLR2 up-related genes in specimens of animals

Figure S3. Optimal dose of TLR2 ligand and HBVs for stimulation

Figure S4. Optimal dose of metabolic inhibitors for blocking experiment

Supplementary Figure Legends

## References

[1] Terrault NA (2018). Update on prevention, diagnosis, and treatment and of chronic hepatitis B: AASLD 2018 Hepatitis B Guidance. Hepatology.

[2] Revill PA (2019). A global scientific strategy to cure hepatitis B. Lancet Gastroenterol. Hepatol..

[3] Cooper A, Tal G, Lider O, Shaul Y (2005). Cytokine induction by the hepatitis B virus capsid in macrophages is facilitated by membrane heparan sulfate and involves TLR2. J. Immunol..

[4] Vanlandschoot P, Van Houtte F, Serruys B, Leroux-Roels G (2007). Contamination of a recombinant hepatitis B virus nucleocapsid preparation with a human B-cell activator. J. Virol..

[5] Vanlandschoot P, Van Houtte F, Ulrichts P, Tavernier J, Leroux-Roels G (2005). Immunostimulatory potential of hepatitis B nucleocapsid preparations: lipopolysaccharide contamination should not be overlooked. J. Gen. Virol..

[6] Zhenhua, Z. et al. Hepatitis B virus particles activate toll‐like receptor 2 signaling initial upon infection of primary human hepatocytes. *Figshare*10.1002/hep.31112 (2020). 10.1002/hep.31112 31925967

[7] Ma Z (2017). The IL-1R/TLR signaling pathway is essential for efficient CD8(+) T-cell responses against hepatitis B virus in the hydrodynamic injection mouse model. Cell. Mol. Immunol..

[8] Geng D (2010). When Toll-like receptor and T-cell receptor signals collide: a mechanism for enhanced CD8 T-cell effector function. Blood.

[9] Liu C, Chapman NM, Karmaus PW, Zeng H, Chi H (2015). mTOR and metabolic regulation of conventional and regulatory T cells. J. Leukoc. Biol..

[10] Qian L (2019). Toll-like receptor 7 activation enhances CD8+ T cell effector functions by promoting cellular glycolysis. Front. Immunol..

[11] Ejuan Z (2019). TLR2 stimulation increases cellular metabolism in CD8+ T cells and thereby enhances CD8+ T cell activation, function, and antiviral activity. J. Immunol..

[12] Zheng T (2015). The monocarboxylate transporter 4 is required for glycolytic reprogramming and inflammatory response in macrophages. J. Biol. Chem..

[13] Nina EM (2020). Glycolytic reprogramming of macrophages activated by NOD1 and TLR4 agonists: no association with proinflammatory cytokine production in normoxia. J. Biol. Chem..

[14] Alice RB (2018). Circulating and intrahepatic antiviral B cells are defective in hepatitis B. J. Clin. Investig..

[15] Zhiyong M, Ejuan Z, Shicheng G, Yong X, Mengji L (2019). Toward a functional cure for hepatitis B: the rationale and challenges for therapeutic targeting of the B cell immune response. Front. Immunol..

[16] Barnaba V, Franco A, Alberti A, Benvenuto R, Balsano F (1990). Selective killing of hepatitis B envelope antigen-specific B cells by class I-restricted, exogenous antigen-specific T lymphocytes. Nature.

[17] Milich DR (1997). Role of B cells in antigen presentation of the hepatitis B core. Proc. Natl Acad. Sci. USA.

[18] Shen P, Fillatreau S (2015). Antibody-independent functions of B cells: a focus on cytokines. Nat. Rev. Immunol..

[19] Waters LR, Ahsan FM, Wolf DM, Shirihai O, Teitell MA (2018). Initial B cell activation induces metabolic reprogramming and mitochondrial remodeling. iScience.

[20] Simon PF (2012). Transcriptomic analysis of the woodchuck model of chronic hepatitis B. Hepatology.

[21] Li S (2014). Molecular signatures of antibody responses derived from a systems biological study of 5 human vaccines. Nat. Immunol..

[22] Shweta J, Sathi BC, Javed NA (2013). Combinatorial signaling through TLR-2 and CD86 augments activation and differentiation of resting B cells. PLoS ONE.

[23] Liu J (2018). TLR2 stimulation strengthens intrahepatic myeloid-derived cell-mediated T cell tolerance through inducing Kupffer cell expansion and IL-10 production. J. Immunol..

[24] Sun D, Nassal M (2006). Stable HepG2- and Huh7-based human hepatoma cell lines for efficient regulated expression of infectious hepatitis B virus. J. Hepatol..

[25] Kosinska AD (2017). Low hepatitis B virus-specific T-cell response in males correlates with high regulatory T-cell numbers in murine models. Hepatology.

[26] Liu J (2013). TLR1/2 ligand–stimulated mouse liver endothelial cells secrete IL-12 and trigger CD8+ T cell immunity in vitro. J. Immunol..

[27] Kawasaki T, Kawai T (2014). Toll-like receptor signaling pathways. Front. Immunol..

[28] Jellusova J, Rickert RC (2017). A brake for B cell proliferation: appropriate responses to metabolic stress are crucial to maintain B cell viability and prevent malignant outgrowth. BioEssays.

[29] Boothby M, Rickert RC (2017). Metabolic regulation of the immune humoral response. Immunity.

[30] Caro-Maldonado A (2014). Metabolic reprogramming is required for antibody production that is suppressed in anergic but exaggerated in chronically BAFF-exposed B cells. J. Immunol..

[31] Cho SH (2011). Glycolytic rate and lymphomagenesis depend on PARP14, an ADP ribosyltransferase of the B aggressive lymphoma (BAL) family. Proc. Natl Acad. Sci. USA.

[32] Doughty CA (2006). Antigen receptor-mediated changes in glucose metabolism in B lymphocytes: role of phosphatidylinositol 3-kinase signaling in the glycolytic control of growth. Blood.

[33] Jung CH, Ro SH, Cao J, Otto NM, Kim DH (2010). mTOR regulation of autophagy. FEBS Lett..

[34] Wullschleger S, Loewith R, Hall MN (2006). TOR signaling in growth and metabolism. Cell.

[35] Katell B (2019). Mimicking immune signatures of flavivirus infection with targeted adjuvants improves dengue subunit vaccine immunogenicity. NPJ Vaccines.

[36] Ahmad FK, Scott MR, Qing L, Henry B, Roxana ER (2017). Toll like receptor 2 engagement on CD4+ T cells promotes TH9 differentiation and function. Eur. J. Immunol..

[37] Suzanne KB, Christine EO’C, Christine AW, Ruaidhrí JC (2018). Toll-like receptors drive specific patterns of tolerance and training on restimulation of macrophages. Front. Immunol..

[38] Matthew ER (2015). limma powers differential expression analyses for RNA-sequencing and microarray studies. Nucleic Acids Res..

[39] Ian TJ, Jorge C (2016). Principal component analysis: a review and recent developments. Philos. Trans. A Math. Phys. Eng. Sci..

[40] Wickham, H. *ggplot2: Elegant Graphics for Data Analysis*. (Springer-Verlag New York, 2016). https://ggplot2.tidyverse.org.

[41] Aravind, S. et al. Gene set enrichment analysis: a knowledge-based approach for interpreting genome-wide expression profiles. *Proc. Natl. Acad. Sci. USA*. **102**, 15545–15550 (2005). 10.1073/pnas.0506580102 PMC1239896 16199517

