# Supplementary figures and images for: Hepatitis B virus particles activate B cells through the TLR2–MyD88–mTOR axis
Source: Cell Death Dis. 2021 Jan 4;12(1):34. doi: 10.1038/s41419-020-03284-1 (PMC7791069; doi:10.1038/s41419-020-03284-1)

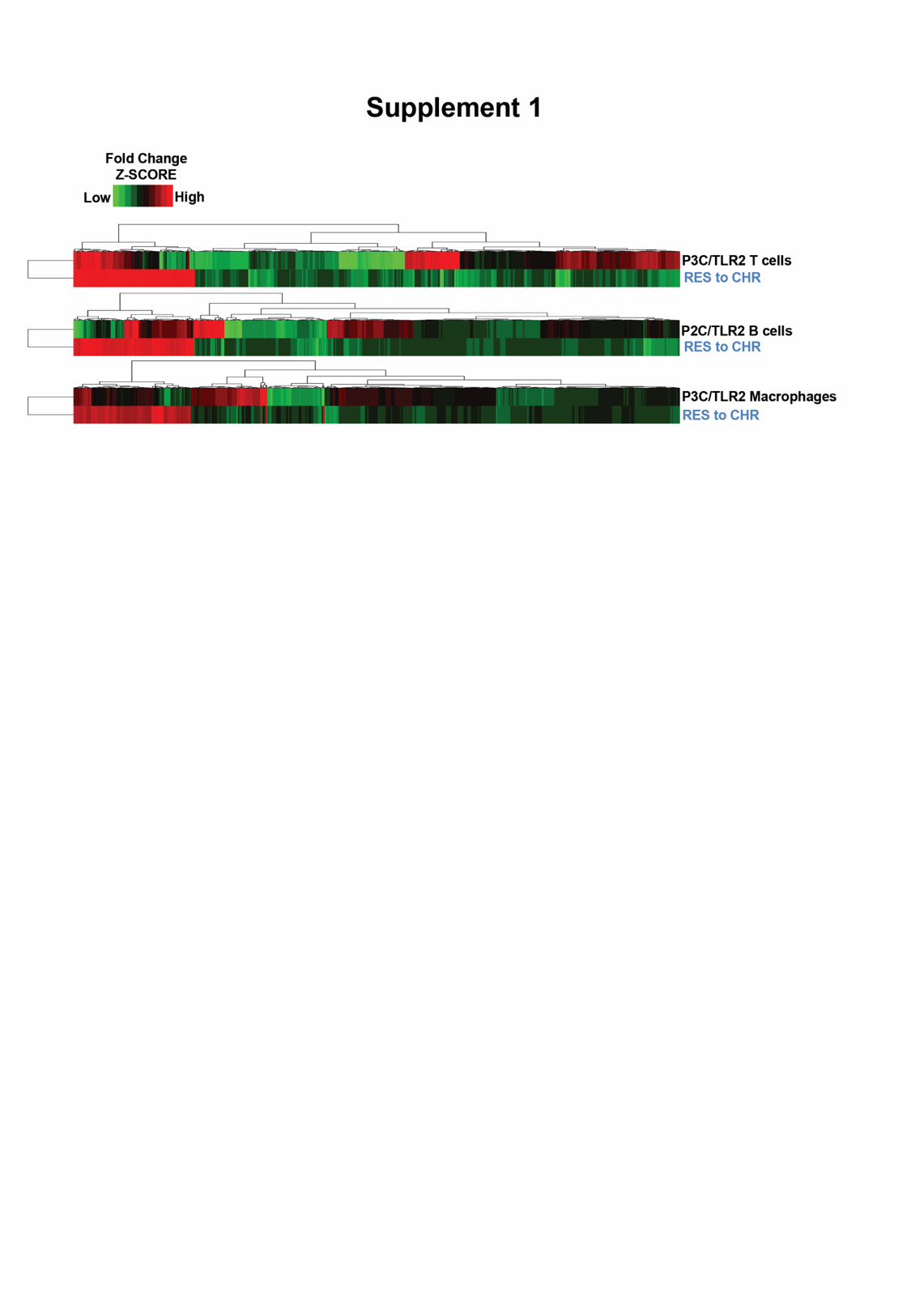

Supplement: Supplementary file 1 — Figure S1. Comparison of TLR2-related signature in specimens of animals [file 41419_2020_3284_MOESM1_ESM.tif]

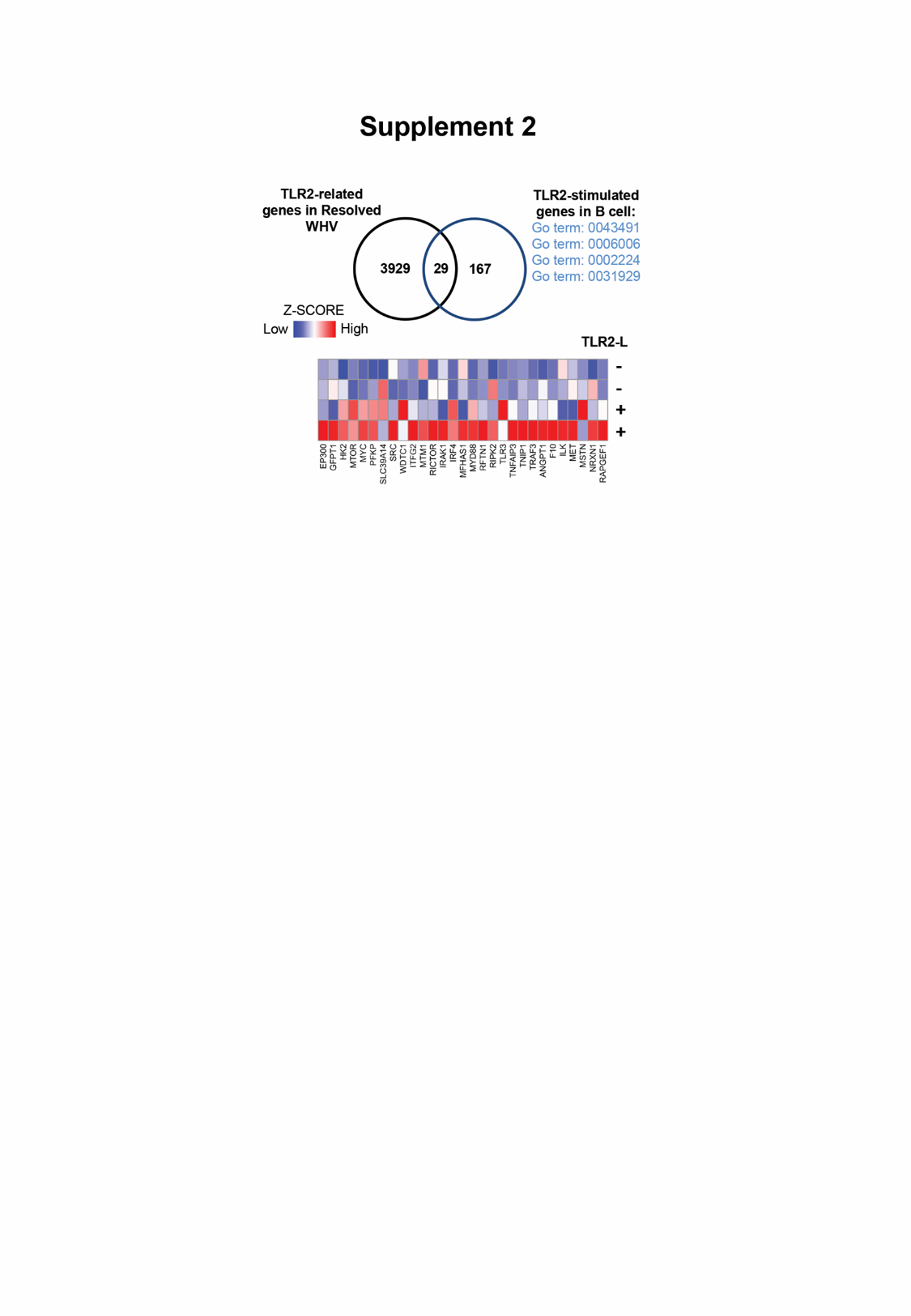

Supplement: Supplementary file 2 — Figure S2. Overlapping of TLR2 up-related genes in specimens of animals [file 41419_2020_3284_MOESM2_ESM.tif]

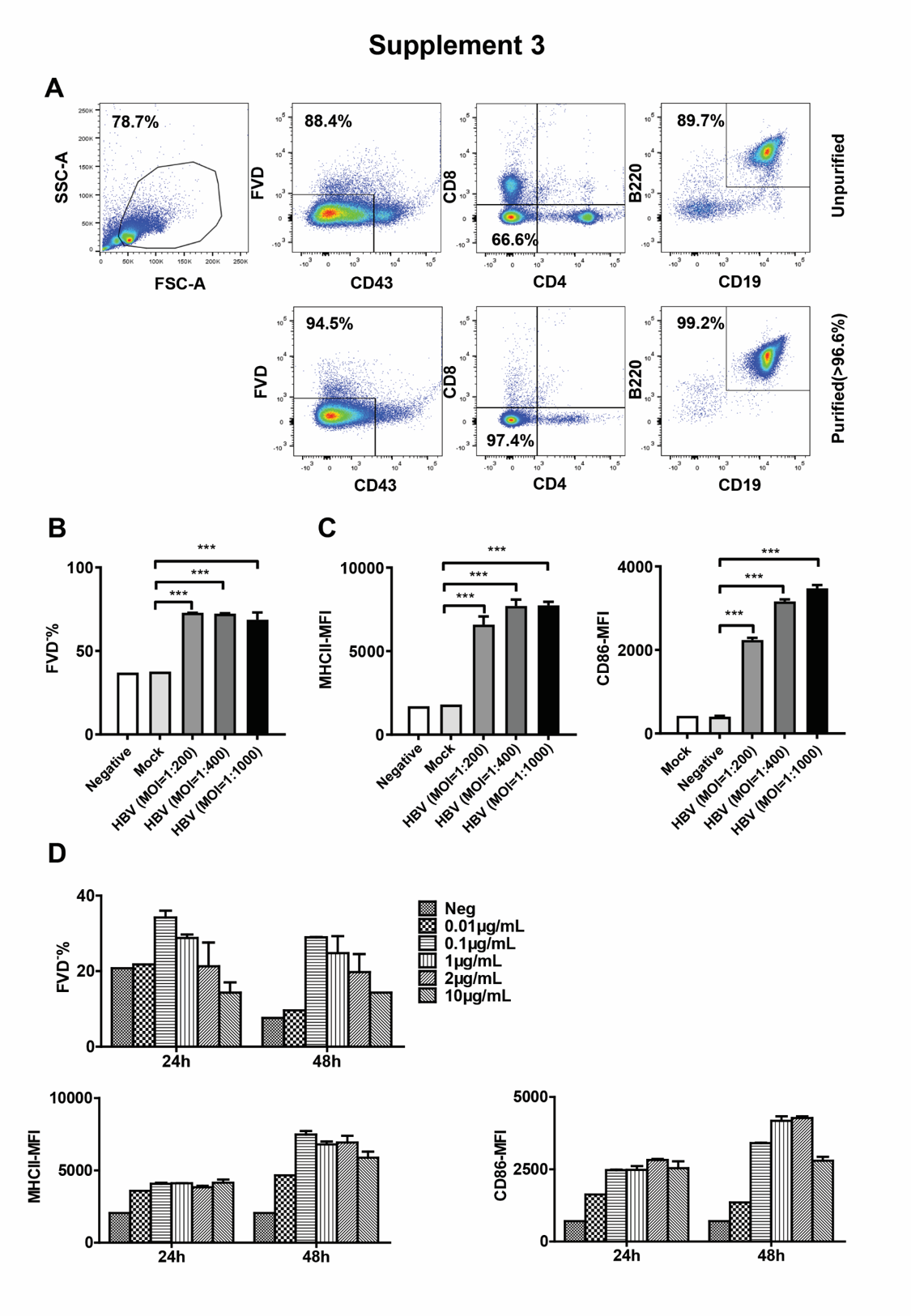

Supplement: Supplementary file 3 — Figure S3. Optimal dose of TLR2 ligand and HBVs for stimulation [file 41419_2020_3284_MOESM3_ESM.tif]

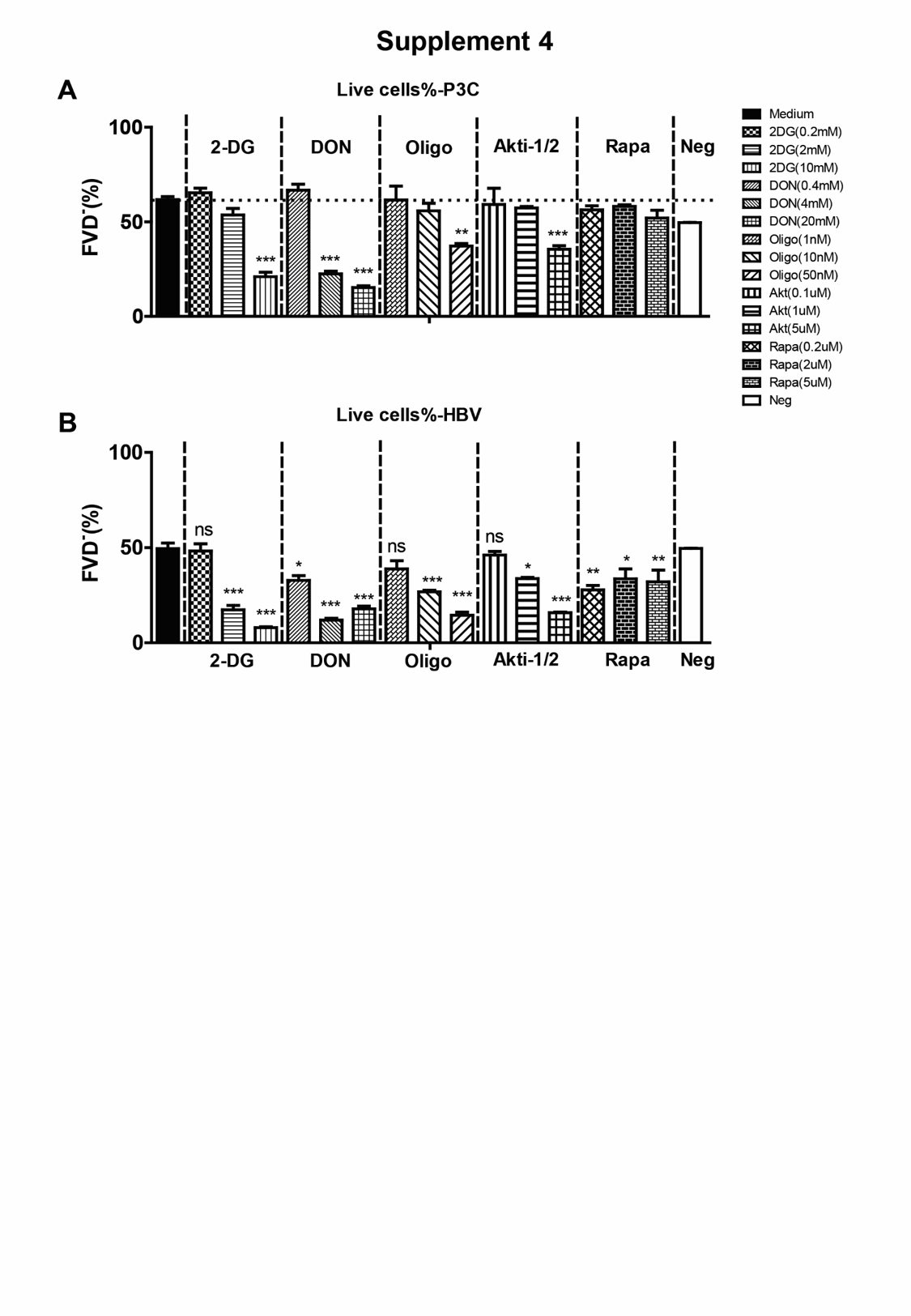

Supplement: Supplementary file 4 — Figure S4. Optimal dose of metabolic inhibitors for blocking experiment [file 41419_2020_3284_MOESM4_ESM.tif]
