# Supplementary material for: Hepatitis B virus particles activate B cells through the TLR2–MyD88–mTOR axis
Source: Cell Death Dis. 2021 Jan 4;12(1):34. doi: 10.1038/s41419-020-03284-1 (PMC7791069; doi:10.1038/s41419-020-03284-1)
Supplement: Supplementary file 5 — Supplementary Figure Legends [file 41419_2020_3284_MOESM5_ESM.docx]

**Supplementary Figure Legends**

**Figure S1. Comparison of TLR2-related signature in specimens of animals**

TLR2-correlated genes in resolved WHV were compared with those in TLR2-stimulated B cells, T cells, and macrophages.

**Figure S2. Overlapping of TLR2 up-related genes in specimens of animals**

TLR2-related genes in resolved WHV samples and 196 upregulated genes included in four GO terms (GO terms: 0006006; 0043491; 0002224; 0031929) were displayed in TLR2-stimulated B cells. Overlapping genes are displayed using a heatmap.

**Figure S3. Optimal dose of TLR2 ligand and HBVs for stimulation**

(A) Gate strategy of purified B cells. (B, C) The purified B cells were stimulated with HBV particles (MOI: 200-1000)/TLR2-L (0.01-10 μg/mL) for 24 hr. (D) Viability of B cells was assessed by FVD staining. Cell activation was assessed by MHCII and CD86 expression.

**Figure S4. Optimal dose of metabolic inhibitors for blocking experiment**

The purified B cells were stimulated with HBV particles (MOI: 1000)/TLR2-L (2 μg/mL) for 24 hr. Doses of metabolic inhibitors such as 2-DG, DON, oligomycin, Akti-1/2 and Rapamycin were added to it. (A) Viability of B cells was assessed by FVD staining in HBVs stimulated B cells. (B) Viability of B cells was assessed by FVD staining in the TLR2-stimulated B cells.
